# Eco-friendly zinc-chitosan/poly(l-lactic acid)/polyurethane nanocomposites for dye removal and antimicrobial wastewater treatment

**DOI:** 10.1038/s41598-025-18431-x

**Published:** 2025-09-15

**Authors:** Magda M. Ragab, Amr M. Beltagi, Samia M. Elsigeny, Shaban Y. Shaban

**Affiliations:** Analytical and Bioinorganic Chemistry Departments, Faculty of Science, Kafrelsheikh University, Kafrelsheikh, 33516 Egypt

**Keywords:** Dye adsorption, Antimicrobial activity, Wastewater treatment, Eco-friendly adsorbents, Sustainable, Reusability, Chemistry, Environmental sciences, Materials science, Nanoscience and technology

## Abstract

This research explores the development of sustainable zinc-chitosan/poly(L-lactic acid)/polyurethane (ZnCS/PLA/PU) nanocomposites for the dual-purpose treatment of wastewater by removing Acid Blue 25 dye and inhibiting pathogens. The nanocomposites were synthesized at ZnCS-to-PU ratios of 4:1 (ZCPP41), 1:1 (ZCPP11), and 1:4 (ZCPP14) and feature particle sizes of 191–365 nm and zeta potentials ranging from + 39.1 to -9.7 mV. ZCPP41 demonstrates an impressive adsorption capacity of 142 ± 1.9 mg/g and 98.8% removal efficiency at pH 2, facilitated by electrostatic interactions below a pHzpc of 5.6, complemented by charge-assisted hydrogen bonding and surface complexation through chitosan’s -OH and -NH₂ groups. Kinetic studies align with the pseudo-second-order model (k₂ = 0.0071 g/mg min, R² = 0.998), while intraparticle diffusion (k_ipd_ = 3.22 to 5.93 mg/g min^0.5^, R² = 0.965) and swelling-enhanced pore filling support deeper dye penetration, corroborated by Langmuir (q_max_ = 142 ± 1.9 mg/g, R² = 0.956) and Freundlich (R² = 0.979) isotherm fits. ZCPP11 exhibits robust antibacterial activity, with inhibition zones of 27.0 mm against Gram-positive bacteria, surpassing gentamicin (24.3 mm), driven by the synergistic effects of Zn²⁺ and chitosan. Desorption with 0.01 M Na₂CO₃ yields 96.5–98.1% efficiency, retaining 71% activity over three cycles, underscoring practical utility. Despite biodegradability, challenges include untested efficacy in complex matrices and scalability. Future work will optimize neutral pH performance and evaluate fixed-bed systems.

**Supplementary Information:**

The online version contains supplementary material available at 10.1038/s41598-025-18431-x.

## Introduction

Water scarcity and pollution pose critical global challenges, exacerbated by the release of synthetic dyes and microbial contaminants into aquatic systems. Emerging pollutants, such as Acid Blue 25, a common anionic dye used in textile, pharmaceutical, and food industries, contribute to reduced oxygen levels and sunlight penetration, severely impacting aquatic ecosystems and human health^1^. The chemical stability and resistance to degradation of these dyes necessitate advanced wastewater treatment strategies^2,3^. Concurrently, microbial contamination by pathogens like *Staphylococcus aureus* and *Escherichia coli* in wastewater heightens public health risks, complicating treatment processes^4^. Traditional adsorbents, such as activated carbon, offer high adsorption capacities (e.g., 500–1000 mg/g for methylene blue) but are limited by high costs, non-biodegradability, and lack of antimicrobial properties^5,6^. This has spurred research into sustainable, multifunctional materials capable of addressing both chemical and biological pollutants.

Adsorption stands out as an efficient, cost-effective treatment method, leveraging materials with high surface areas and functional groups to remove dyes and pathogens. Biopolymers, including chitosan, have emerged as promising alternatives due to their biodegradability and reactivity^7–9^. Chitosan, derived from chitin, contains hydroxyl and amino groups that facilitate adsorption of anionic dyes under acidic conditions (e.g., 267 mg/g for Food Red 17 with chitosan-based composites)^10,11^. Recent studies have advanced chitosan-based adsorbents, such as chitosan-montmorillonite composites achieving 98.5% dye removal efficiency^12^, and chitosan-supported CoFe₂O₄ showing enhanced removal of anthraquinone dyes^13^. However, its mechanical weakness and limited affinity for certain pollutants restrict standalone use^14^. Blending chitosan with biodegradable polymers like poly(L-lactic acid) (PLA) and polyurethane (PU)—the latter being a biodegradable polyester-based variant in this study—enhances mechanical stability and sustainability^15,16^. For instance, PU/chitosan composites have demonstrated dye adsorption capacities up to 201.9 mg/g for Reactive Black 5^17,18^, while PLA/chitosan blends improve hydrolytic degradability and tensile energy absorption^19^. Chitosan-based hydrogels have also shown promise, with capacities up to 200 mg/g for methylene blue under optimized conditions^20^. Despite these advances, few studies explore Zn(II)-chitosan complexes integrated with PLA/PU matrices for dual-functional applications.

Modification of biopolymers with metal ions, such as zinc(II), boosts adsorption and antimicrobial properties by forming complexes that bind pollutants and disrupt bacterial cell walls^21,22^. This study hypothesizes that ZnCS/PLA/PU nanocomposites, synthesized at varying ZnCS-to-PU ratios, can simultaneously remove Acid Blue 25 dye and control pathogens, leveraging chitosan’s functional groups, PLA/PU’s biodegradability, and Zn(II)’s synergistic effects. The methodology involves synthesizing nanocomposites (ZCPP41, ZCPP11, ZCPP14) via crosslinking with tripolyphosphate (TPP) and electrostatic integration, followed by characterization (FTIR, SEM, DLS, zeta potential) and evaluation of adsorption kinetics, isotherms, antibacterial activity, and reusability. By addressing these objectives, this work aims to develop an eco-friendly, cost-effective solution for wastewater remediation, with potential for optimization in complex matrices. The novelty of this work lies in the integration of Zn(II)-chitosan complexes with biodegradable PLA/PU matrices at varying ratios, enabling dual functionality (dye adsorption and antimicrobial activity) in a single eco-friendly nanocomposite, which few studies have explored.

## Materials and methods

### Materials

CS (C₅₆H₁₀₃N₉O₃₉) (low molecular weight, degree of deacetylation 70–90%), zinc acetate (> 98%), and polylactic acid (PLA, C₃H₅O₃, molecular weight 20,000–50,000 g/mol, ≥ 98% purity) were purchased from Sigma-Aldrich. The polyurethane (PU) used is a biodegradable polyester-based polyurethane (molecular weight 20,000–50,000 g/mol, ≥ 98% purity, Sigma-Aldrich), selected for its hydrolyzable ester bonds that enable degradation under specific environmental conditions. All chemicals and reagents used in this study were of analytical grade.

### Adsorbent preparation

The ZnCS was prepared as follows: dissolved CS (0.34 g) into 1% acetic acid (100 mL) was agitated for 24 h at room temperature at a speed of 180 rpm to achieve a thoroughly homogenous solution. Then 0.1 g of zinc acetate dissolved into 50 mL of water was slowly added into the CS solution while continuously stirring until a homogenous solution was achieved. PU solution was prepared by dissolving a calculated amount of PU in DMF solution and mixed with PLA solution (0.1 g in 10 ml dichloromethane) and stirred at room temperature for 30 min. The PLA/PU mixture was poured into Zn(II)CS solution with different contents for proper adsorption through the electrostatic interaction. ZnCS to PU component ratios were 4:1 (w/w) (ZCPP41 NCs), 1:1(w/w) (ZCPP11 NCs) and 1:4 (w/w) (ZCPP14 NCs). After adding glycerol as a plasticizer, magnetic stirring was used to homogenize the mix solution overnight. 5 ml of TPP (0.6 mg/ml water) was added into the above emulsion drop wise with stirring. To facilitate more cross-linking of the NPs, the solution was further stirred for 24 h. Finally, the particles were collected by centrifugation at 12,000 rpm for 10 min. These particles were further purified by washing several times with deionized water followed by centrifugation. Finally these particles were dried at 60 ◦C and were designated as ZCPP41 NCs, ZCPP11 NCs and ZCPP14 NCs.

### Characterization techniques

An FT-IR spectrometer (Perkin Elmer, Germany) was used to record spectra throughout a 400–4000 cm^–1^ range in order to evaluate the functional group linkage in the prepared compounds. Using the dynamic light scattering (DLS) method on a zetasizer (Malvern Instruments, 1000 HS, Malvern, UK) in tris(hydroxymethyl)aminomethane (a tris-buffer solution), the polydispersity index (PDI) of the diluted solution was determined. Using a laser wavelength of 632 nm and a predetermined scattering angle of 173°, DLS with noninvasive back scattering (DLS-NIBS) was utilized to calculate the hydrodynamic diameter of the samples, assuming a spherical form. A combination of mixed laser Doppler electrophoresis and phase analysis light scattering (M3-PALS) was used to measure the zeta potential (ζ) in a tris-buffer solution. Both measurements were performed using the Malvern Zetasizer Nano, Model ZS 3600 (Malvern Instruments, Worcestershire, UK). SEM technology (Seron Technology, South Korea) was used to examine the morphology of the prepared sample. To examine optical properties, a UV-vis spectrophotometer (Model Per-kin-Elmer Lambda) was utilized.

### Adsorption studies

Working standard solutions were subsequently made from the stock solution by diluting precisely weighed solutes until the stock solution comprised 1 g of the Acid Blue 25 dye equivalent in 1 L of distilled water. Before the adsorption investigation, an aliquot of H_2_SO_4_ or NaOH was added to the working solution to maintain its pH. The investigations on batch equilibrium and kinetics adsorption were carried out in a batch method using plastic flasks holding 25 mL of Acid Blue 25 dye solutions with an adsorbent concentration range of 0.4 g/L and 10–50 mg/L. Samples were taken at predetermined intervals (0–240 min), and a UV–vis spectrophotometer (Peak Instruments Inc. C-7200 (USA) UV–visible spectrophotometer) was used to measure the Acid Blue 25 dye concentrations in aqueous media at λ_max_ = 600 nm. Equations 1–3 below were used to estimate the amount of Acid Blue 25 dye adsorbed (mg/g) by the adsorbents as a function of time (*Q*_t_) and at equilibrium (*Q*_e_) as well as the removal efficiency R (%).

1 $$\:{Q}_{t}=\frac{{(C}_{0}-{C}_{t})\:x\:V}{m}$$

2 $$\:{Q}_{e}=\frac{{(C}_{0}-{C}_{e})\:x\:V}{m}$$

3 $$\:Removel\:\left(\%\right)=\frac{{(C}_{0}-{C}_{t})\:x\:100}{{C}_{0}}$$

Where *C*_o_, *C*_t_ and *C*_e_ are the initial, time *t* and equilibrium concentrations (mg L^− 1^) of the Acid Blue 25 dye respectively, V is volume (*L*) of the solution and m is the mass (*g*) of the adsorbent.

### Desorption studies

Adsorbents with high reusability are always used in practical applications. Several desorbing agents were created in this study to extract Acid Blue 25 dye from the NCs. First, 100 mL (100 mg/L) of Acid Blue 25 dye was added to 30 mg of ZCPP41, ZCPP11, and ZCPP14 NCs at a fixed pH of 2.0. The ZCPP41, ZCPP11, and ZCPP14 dye-adsorbed NCs were immersed in a 0.01 M aqueous solution of HCl and Na_2_CO_3_ at a 12 mg/40 mL (S/L) ratio. Equations (4) and (5) display the amount of desorption and the rate of desorption efficiency, respectively.

4 $$\:{q}_{des}=\frac{{(C}_{1})\:x\:V}{M}$$

5 $$\:\:\%\:desorption=\frac{{q}_{des}\:x\:V}{{q}_{e}}$$

where C_1_ is the dye concentration of the desorption supernatant (mg/L), V is the volume of the desorption solution (L), M is the adsorbent mass (g), *q*_des_ is the amount of dye that is desorbing (mg/g), and %desorption is the rate of desorption efficiency (%).

### Swelling studies

The swelling behavior was investigated using a gravimetric approach. The ZCPP41, ZCPP11, and ZCPP14 NCs were allowed to swell in Acid Blue 25 dye solution (100 mg/L) with a pH of 2.0 at room temperature. The swelling of each sample was monitored at a certain period after the extra surface water had been thoroughly removed with moist filter paper. The swelling ratio was obtained from the swollen weight (*W*_w_) and the dry weight (*W*_d_) of the NCs as follow:

6 $${\text{Swelling ratio }}\left( {{\text{g}}/{\text{g}}} \right){\text{ }}={\text{ }}({W_{\text{w}}} - {W_{\text{d}}})/{W_{\text{d}}}$$

### Point of zero discharge (ZPC)

The point of zero charge for the sorbent materials was determined^22,23^. A stock solution of 0.01 M NaCl was prepared and divided into five 125 mL Erlenmeyer flasks, each containing 25 mL. The pH of these solutions was adjusted to span values between 2 and 10 using NaOH/HCl, ensuring each flask had a distinct pH level. Approximately 100 mg of sorbent material was added to each solution. After equilibrating for 48 h, the final pH was measured in each flask. A plot of final versus initial pH revealed the intersection point, which corresponded to the point of zero charge (pHzpc) for each material.

### Antibacterial activity

Antibacterial activity of nanocomposites was assessed against Gram-positive (*B. subtilis*, *E. faecalis*, *S. aureus*) and Gram-negative (*E. coli*, *K. pneumoniae*, *S. typhi)* bacteria using the agar well diffusion method. Agar plates were inoculated with microbial inoculum, and wells (6–8 mm) were punched using a sterile cork borer. Wells were filled with 20–100 µL of ZCPP41, ZCPP11, and ZCPP14 NCs solution. Plates were incubated for 24 h, and inhibition zone diameters (mm) were measured.

## Results and discussions

### Synthesis and characterization of zncs/pla/pu nanocomposites

The ZnCS/PLA/PU NCs were obtained in two stages as follows: The first (i) step consisted of the synthesis of the PU in different weights with the soft segment PLA. In the second (ii) step, the ZnCS/PLA/PU was obtained by the crosslinking reaction of the PU/PLA with ZnCS using TPP as the crosslinking agent. ZnCS to PU component ratios were 4:1 (w/w) (ZCPP41 NCs), 1:1 (w/w) (ZCPP11 NCs), and 1:4 (w/w) (ZCPP14 NCs). The synthesis mechanism involves a multi-step process where the amino groups (-NH₂) of chitosan in ZnCS form ionic complexes with the phosphate groups of TPP, creating a stable three-dimensional network that encapsulates Zn(II) ions, enhancing both adsorption sites and antimicrobial properties. Subsequently, the PU/PLA matrix, featuring biodegradable polyester-based PU and PLA’s negatively charged carboxyl groups, integrates with the ZnCS-TPP network through electrostatic interactions, with PU’s hydrolyzable ester bonds contributing to the composite’s flexibility and degradability.

#### IR analysis

**Fig. 1:**
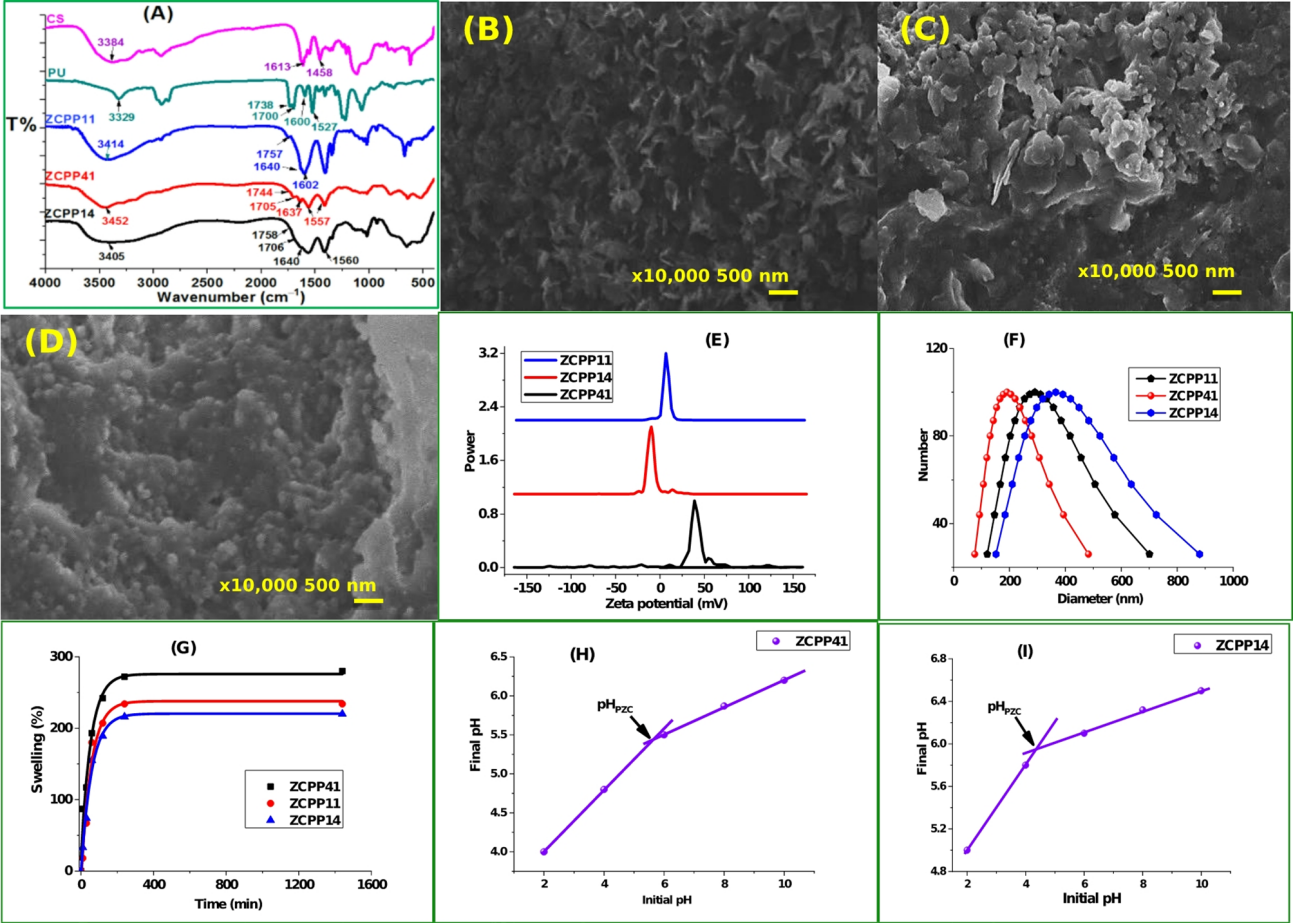
Characterization of ZnCS/PLA/PU nanocomposites. (A) FT-IR spectra (400–4000 cm⁻¹) of ZCPP41, ZCPP11, and ZCPP14 NCs, highlighting functional group vibrations; SEM images of (B) ZCPP41, (C) ZCPP11, and (D) ZCPP14 NCs (scale bars: 500 nm, magnification: ×10,000), showing surface morphology; (E) Zeta potential distribution (+ 39.1 to -9.7 mV); (F) Particle size distribution diagram (191–365 nm) via DLS; (G) Equilibrium swelling degrees in water at 298 K and point of zero charge (pHzpc) values for (H) ZCPP41 (pHzpc ≈ 5.6) and (I) ZCPP14 (pHzpc ≈ 4.3) sorbents, indicating surface charge properties.

The FT-IR spectra of CS, PU, ZCPP41 NCs, ZCPP11 NCs, and ZCPP14 NCs are displayed in Fig. 1A. Pure PU exhibited a peak at 3329 cm^− 1^ due to urethane’s H-bonded stretching (−NH). The bands at 1748 and 1700 cm^− 1^ corresponded to free and hydrogen-bonded carbonyl groups. The peaks at 1600 cm^− 1^ (C = O stretching), 1527 cm^− 1^ (NH bending of HNCOO), 1464 cm^− 1^ (CH_2_ bending), 1247 cm^− 1^ (COC stretching), and 1069 cm^−1^ (CO stretching) were similarly diagnostic of PU^24,25^. The typical absorption peaks of CS were found at 3384 cm^−1^ (−OH group), 2934, 2857 cm^−1^ (asymmetric stretching vibration of CH_2_ & CH group), 1613 cm^−1^ (C=O stretching of amide group), 1458 cm^−1^ (−NH bending of NH_2_ group), 1338 cm^−1^ (stretching vibrations of amide III group), 1149, 1110, and 1036 cm^−1^ (saccharide structure)^26,27^. The distinct spectral peaks of both CS and PU changed when they were combined due to the physical and chemical interactions. For instance, in the ZCPP11 nanocomposite’s spectrum (Fig. 1A), the CS stretching band at 3384 and the PU peak at 3329 both shifted to 3287 and 3414 cm^−1^, respectively, and both bands grew wider, suggesting that PU and CS have intermolecular hydrogen bonds^28^. The free carbonyl stretching absorption peak in PU (1739 cm^−1^) moved to a higher wave number (1757 cm^−1^), suggesting that a hydrogen bond formed between the C=O group (PU) and the −OH group at CH_2_OH (CS)^29^. The nanocomposites exhibited a considerable shift in the typical peak at 1600 cm^−1^ (Fig. 1A) to a higher wave number (1640 cm^−1^), indicating the synthesis of urea group due to the interaction between the amine group of CS and the terminal NCO group of polyurethane. The ZCPP11 NCs (Fig. 1A) had PU’s typical peaks as well as absorption peaks at 1602 and 1574 cm^−1^, which corresponded to the amide peaks of CS and demonstrated the miscibility of PU & CS. The (−NH) peaks of PU in ZCPP41 NCs, ZCPP11 NCs, and ZCPP14 NCs have been further moved towards higher wave number and have become broader, according to a comparison of PU, CS, ZCPP41 NCs, ZCPP11 NCs, and ZCPP14 NCs spectra (Fig. 1A)^30,31^.‬‬‬‬‬‬‬‬‬‬‬‬‬‬‬‬‬‬‬‬‬‬‬‬‬‬‬‬

#### Morphology and structure

Figures 1B–D display the surface morphology of the produced ZnCS/PLA/PU nanocomposites, analyzed via SEM. The ZCPP41 NCs exhibit a rough, uneven surface with flake-like features within a polymeric matrix, reflecting the nanocomposite’s dispersed nanostructure. These structural characteristics, observed with scale bars of 500 nm and magnification of x10,000, facilitate access for oxyanions to internal functional adsorptive sites. The intraparticle diffusion theory, detailed in the kinetics modeling (“Desorption studies”), supports a second-phase adsorption process enabled by this morphology. As the PU content increases in ZCPP11 NCs, minor agglomeration and surface roughening occur, attributed to the mixing of PLA and PU, which induces characteristic phase separation morphologies indicative of polymer entanglement and interactions. In ZCPP14 NCs, with further increased PU, homogeneous particles without agglomeration emerge, enhancing the general characteristics of the nanocomposite films through uniform dispersion^30^. Post-adsorption SEM images (Figs. 1B–D) reveal surface alterations, such as increased roughness or coverage, confirming dye interaction, while the material is confirmed as a nanocomposite with dispersed nanostructures, not a core-shell structure^31^.

#### Particle size and zeta potential

One crucial factor that affects stability, dispersion, and interaction in different settings is the zeta potential. The composition and ratio of the materials utilized affect the zeta potential, which represents the electro kinetic potential at the sliding plane of particles in a colloidal system. For colloidal systems, stability against aggregation is generally indicated by a larger zeta potential (greater than + 30 mV). In contrast, a zeta potential below this threshold signals potential instability and a tendency for particles to agglomerate^32,33^. By measuring their hydrodynamic sizes and zeta potential values, the **ZCPP41**, **ZCPP11**, and **ZCPP14** nanocomposite particles’ changes in particle sizes and surface charges were examined. The results presented in Table 1; Figs. 1E, F show that as the ratio of chitosan to polyurethane in the nanocomposite varies, there are notable changes in both particle size and surface charge. A reduction in the proportion of CS, which carries a positive charge due to its amino groups, leads to increase particle size. The overall zeta potential of the composite, on the other hand, decreases when the amount of PU, which may have a lower charge, is increased. No precipitation was seen during the synthesis of ZCPP11 and ZCPP14 nanocomposites, even though the surface charges decreased from + 39.1 mV for ZCPP41 to around + 6.8 mV for ZCPP11 and − 9.7 mV for ZCPP14. After a few days, a small aggregation of ZCPP14 particles was observed in the solution. The aggregates of ZCPP14 particles leads to bigger particle size (365 nm) compared to ZCPP41 (191 nm) and ZCPP11 (290 nm). However, these aggregates can be readily re-dispersed into a stable colloidal system through sonication.

**Table 1:** Mean particle diameter and zeta potential values of ZCPP41, ZCPP11, and ZCPP14 nanocomposites, determined by dynamic light scattering (DLS) and zeta potential analysis, respectively.

	Size (nm)	PDI	Zeta potential (mV)
ZCPP41 NCs	191 ± 7	0.391 ± 0.101	+ 39.1 ± 1.8
ZCPP11 NCs	290 ± 20	0.351 ± 0.093	+ 6.8 ± 0.4
ZCPP14 NCs	365 ± 30	0.50 ± 0.108	−9.7 ± 0.8

#### Equilibrium swelling in water

The swelling characteristics of ZCPP41, ZCPP11, and ZCPP14 in PBS at 25 °C were evaluated over a 24-hour period (Fig. 1G). The swelling ratio of all three materials generally increased over time, likely due to the presence of hydrophilic groups in the CS and PLA polymer chains. ZCPP41 demonstrated the most pronounced swelling, its behavior strongly influenced by its CS concentration. All three nanocomposites swelled rapidly in the initial stages, stabilizing after roughly 4 h. The maximum swelling degrees observed were 276% for ZCPP41, 234% for ZCPP11, and 220% for ZCPP14. ZCPP14 exhibited the least swelling relative to the other two composites which may stem from a weaker tendency for solvents to penetrate the fiber domains of CS and PLA, potentially due to hydrogen bonding effects. The high proportion of CS in ZCPP41 promotes significant cross-linking, leading to the formation of defect sites and micropores within the polymer matrix, and ultimately increasing swelling and sorption affinity^10^.

#### Point of zero charge (PZC)

The point of zero charge (pHzpc) indicates the pH at which the ZnCS/PLA/PU nanocomposites exhibit a net zero surface charge, influencing their adsorption behavior^34^. Experimental results show the pHzpc values as 5.6 for ZCPP41, 5.0 for ZCPP11, and 4.3 for ZCPP14, determined from zeta potential measurements (Fig. 1H and I, and Figure S1). These values suggest that under acidic conditions (pH < pHzpc), the sorbent surfaces become positively charged, favoring the adsorption of anionic species like Acid Blue 25, as observed at pH 2 with 98.8% removal efficiency. The most negative zeta potential in ZCPP14, requiring more acidic conditions for neutralization, correlates with its pHzpc of 4.3. This pH-dependent behavior enhances cationic species adsorption above pHzpc, though the focus here remains on anionic dye removal under acidic conditions.

### Adsorption studies

In this section, the influences of CS addition, adsorbent mass, initial pH and initial dye concentration on the dye adsorption were investigated.

#### Effect of pH

**Fig. 2:**
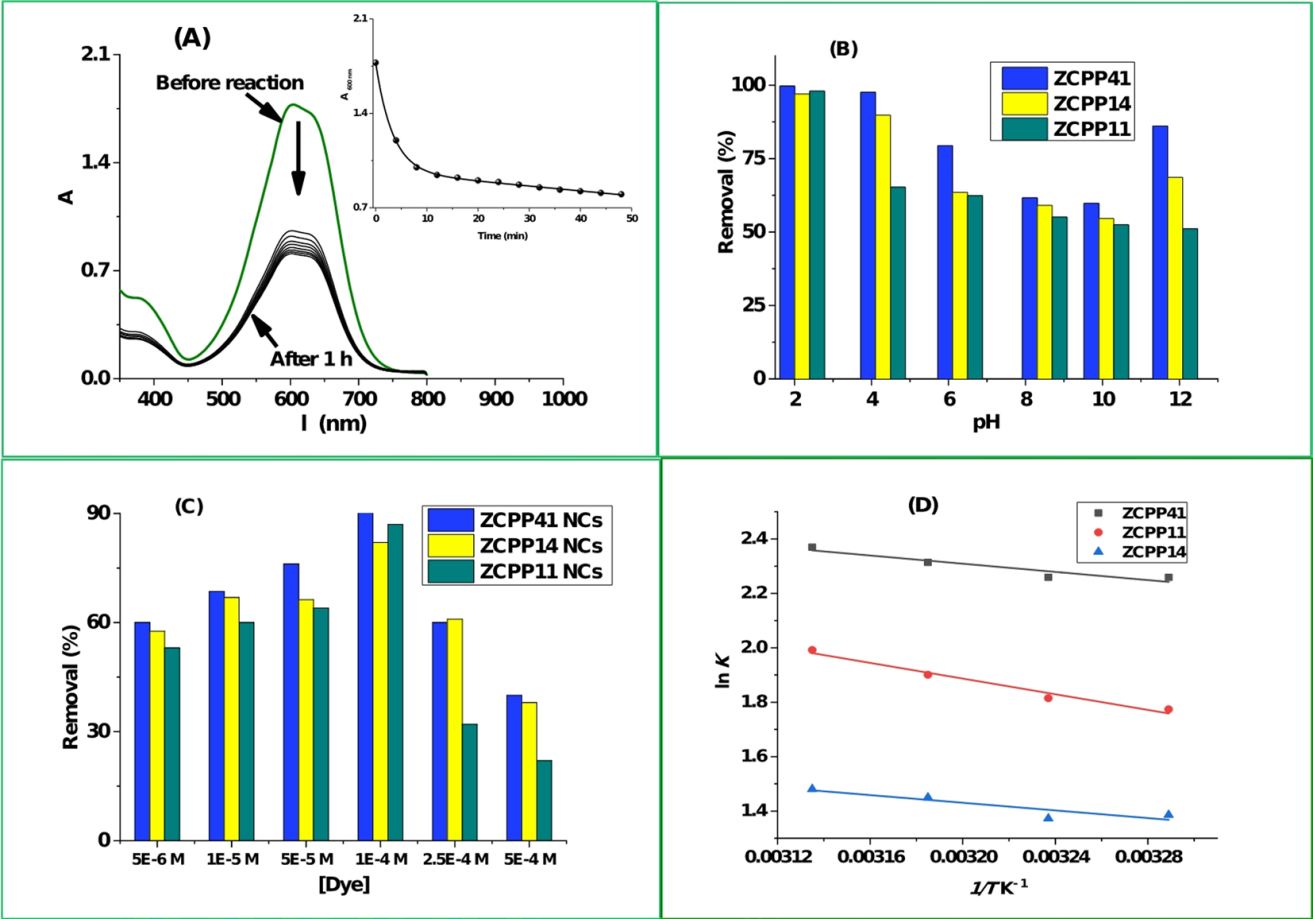
(**A**) UV-Vis spectra of ZCPP41 NCs reacting with Acid Blue 25 dye (inset: absorbance at 600 nm over time); (**B**) effect of pH on adsorption efficiency (pH 2–12); (**C**) influence of initial dye concentration on adsorption capacity (5 × 10⁻^6^ to 5 × 10⁻^4^ M); (D) Van’t Hoff plots of lnK vs. 1/T for ZCPP11, ZCPP41, and ZCPP14 NCs (304–319 K), with linear fits.

One of the key elements that affect how well the process works is the adsorption medium’s pH. It controls the physical and chemical interactions between the adsorbent and the adsorbate in solution by determining their chemical composition and characteristics. A chemical interaction between the positively charged adsorbent and the anionic form of the Acid Blue 25 dye in the acidic medium is suggested by Fig. 2A. Figure 2B demonstrates that the adsorption efficiency of the dye by the three catalysts is pH dependent. The highest removal efficiency of 99.7% (ZCPP14 NCs), 98.8% (ZCPP41 NCs), and 97.0% (ZCPP11 NCs) were obtained at pH 2. Additionally, these observations revealed that the basic medium strongly favors dye desorption. ZCPP14 NCs’s removal efficiency dropped sharply from 99.7 to 54.6%, ZCPP41 NCs’s from 98.8 to 52.5% and ZCPP11 NCs’s from 97.0 to 59.0% when the pH was raised from 2 to 10. The elimination efficiency sharply begins to somewhat increase once again beyond this pH range. Similar to reactions where the mechanism is related to the physisorption process^35^, the adsorption mechanism of the negatively charged anionic dye in the PU chitosan-coated can be associated to the electrostatic interaction between the dye’s negative groups and the adsorbent’s positive sites^36–39^. The protonation of the chitosan amino groups causes the PU chitosan-coated material to become positively charged, which gives the adsorbent a polycationic behavior and increases the electrostatic interaction between the sulfonated groups from Acid Blue 25 dye^40,41^. This is linked to the increase in the maximum adsorption capacity when the pH is acidic. The optimal pH for further adsorption experiments was found to be 2.0.

#### Effect of adsorbent dosage

The influence of adsorbent dosage on the percentage of dye removal is illustrated in Figure S2A. This figure demonstrates that the amount of adsorbent significantly affects adsorption efficiency; specifically, increasing the adsorbent dosage results in a marked enhancement in adsorption performance. As shown in Figure S2A, when the adsorbent mass was raised from 0.001 g to 0.02 g, the percentage of dye removal improved from 60 to 98% for ZCPP41 NCs, from 55 to 88% for ZCPP11 NCs, and from 42 to 65% for ZCPP14 NCs. This trend can be attributed to the direct correlation between adsorbent mass and the availability of active sites for interactions between the adsorbent and the dye molecules. Furthermore, it was determined that incorporating chitosan into the synthesis of polyurethane nanocomposite effectively enhances the material’s adsorption capabilities. The addition of chitosan introduces extra hydroxyl (OH) and amino (NH_2_) groups, which serve as additional adsorption sites, thereby increasing the removal efficiency of Acid Blue 25 dye from the solution.

#### Effect of the initial dye concentration

Figure 2C illustrates that the sorbents ZCPP41 NCs, ZCPP11 NCs, and ZCPP14 NCs were particularly effective in removing initial concentrations of Acid Blue 25 dye ranging from 5 × 10⁻^6^ to 5 × 10⁻⁴ M. However, their effectiveness in removal diminishes as the concentrations of Acid Blue 25 exceed 10⁻⁴ M. Initially, the removal efficiency increased significantly, but it then declined as the initial dye concentration rose. This reduction in dye removal effectiveness at higher initial concentrations may be attributed to the limited number of adsorptive sites available on a constant amount of adsorbent, which becomes saturated. At higher initial dye concentrations, there are more dye molecules to occupy the available adsorption sites on the PU/chitosan surface, and consequently the coverage fraction is higher^36^. Additionally, equilibrium was reached more rapidly in solutions with lower dye concentrations compared to those with higher concentrations, likely due to stronger adsorption driving forces and reduced repulsive interactions between the dye anions in the solid and bulk phases.

#### Effect of temperature

The efficiency of dye adsorption on NCs was measured at various temperatures and is illustrated in Figure S2B. It is evident that the adsorption capacity showed a slight increase as the temperature rose from 298 to 320 K, suggesting an endothermic process. According to Dotto et al., this phenomenon can be attributed to the swelling effect of NCs, which becomes more pronounced at higher temperatures and allows dye molecules to penetrate more deeply into the adsorbent’s structure^10,42^. Thermodynamic parameters were evaluated using the van’t Hoff equation based on temperature-dependent removal efficiencies, as summarized in Table 2. For ZCPP41 NCs, ΔH∘ was 6.26 kJ/mol (endothermic) and ΔS∘ was 39.24 J/mol·K, with ΔG∘ -5.67 kJ/mol at 304 K, indicating spontaneous adsorption. Similar trends were observed for ZCPP11 (ΔH^∘^ = 11.64 kJ/mol, ΔS∘ = 51.55 J/mol.K, ΔG∘ = −4.03 kJ/mol) and ZCPP14 (ΔH∘ = 5.85 kJ/mol, ΔS∘ = 30.76 J/mol.K, ΔG∘ = −3.50 kJ/mol), confirming the process’s favorability. The superior thermodynamic profile of ZCPP41, likely due to its higher chitosan content, underscores its enhanced adsorption efficiency.

**Table 2:** Thermodynamic parameters for the sorption of acid blue 25 on ZCPP ncs.

T (K)	∆G° (kJ/mol)			∆H° (kJ/mol)	∆S° (J/mol·K)
	ZCPP11	ZCPP41	ZCPP14		
304	-4.03	-5.67	-3.50	11.6 (ZCPP11) 6.3 (ZCPP41) 5.9 (ZCPP14)	51.6 (ZCPP11) 39.2 (ZCPP41) 30.8 (ZCPP14)
309	-4.29	-5.87	-3.65		
314	-4.55	-6.07	-3.81		
319	-4.80	-6.26	-3.96		

#### Kinetic results

**Fig. 3:**
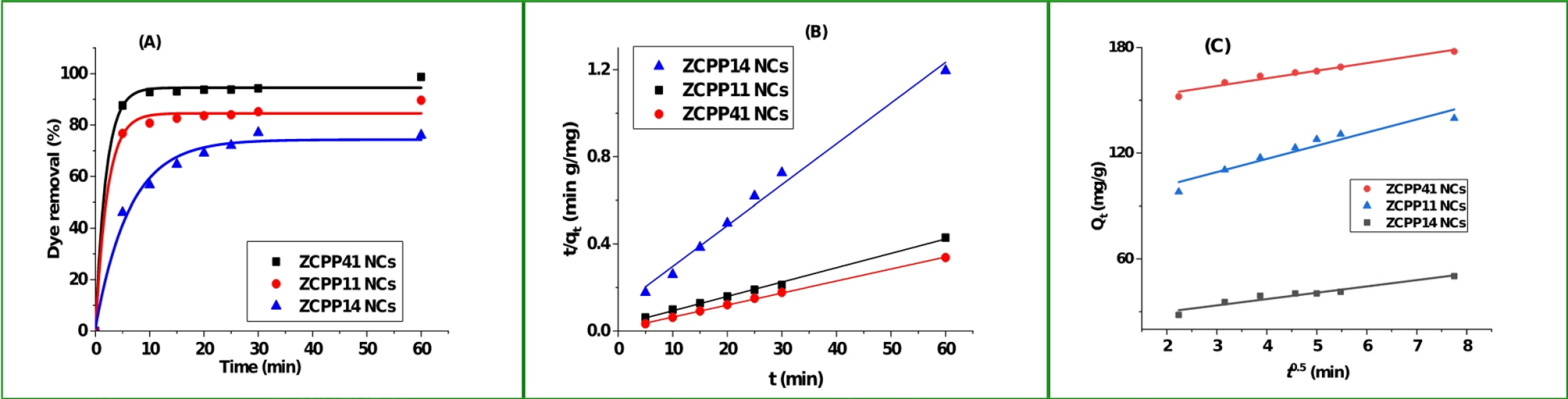
Kinetic analysis of Acid Blue 25 adsorption on ZnCS/PLA/PU nanocomposites at pH 2 and 298 K. (**A**) Adsorption efficiency over time for ZCPP41, ZCPP11, and ZCPP14 NCs; (**B**) pseudo-second-order (PSO) model fit (t/qt vs. t), showing R² values > 0.98; (**C**) intraparticle diffusion (IPD) model fit (q_t_ vs. t^0.5^).

The kinetic behavior of dye adsorption on ZnCS/PLA/PU nanocomposites (NCs) was analyzed by monitoring adsorption efficiency over time (t), as shown in Fig. 3A. Adsorption occurred rapidly within the first 5 min, followed by a gradual increase in capacity between 5 and 20 min. After 60 min, the rate declined significantly, reaching equilibrium for all NCs as surface sites became increasingly occupied^43^. Increasing the chitosan (CS) to polyurethane (PU) ratio from 1:4 to 4:1 enhanced the adsorption capacity (Fig. 3B), attributed to additional hydroxyl (OH) and amino (NH₂) groups from chitosan, providing more adsorption sites and boosting removal efficiency. The kinetic data were analyzed using the pseudo-first-order (PFO) (Figure S3) and pseudo-second-order (PSO) models (Fig. 3B), with Eqs. 7 and 8^44^, respectively.

7 $$log(q_e- q_t) = log(q_e) - k_1 t$$

8 $$\:\frac{t}{{q}_{t}}=\frac{1}{\:{K}_{2}{{q}_{e}}^{2}}+\frac{1}{{q}_{e}}t$$

9 $${q_t}={\text{ }}{k_{ipd}}{t^{0.5}}+{\text{ }}C$$

Results are summarized in Table 3. For ZCPP41 NCs, the adsorption capacity was 174.3 ± 1.9 mg/g calculated by the PSO model (R² = 0.998), compared to and 118.31 mg/g by the PFO model (R² = 0.933), indicating better fit with PSO, suggesting chemisorption as the rate-limiting step. The PSO parameters q_e_ and k₂ were derived from the t/qt vs. t plot (Fig. 3B), with k₂ decreasing to 0.0025 g/mg min as the CS: PU ratio shifted from 4:1 to 1:4. Intraparticle diffusion (IPD) modeling, described by Eq. 9, where q_t_ is the amount adsorbed (mg/g), k_ipd_ is the rate constant (mg/g min^0.5^), and C is the intercept (mg/g) reflecting boundary layer effects, was analyzed using Fig. 3C. IPD modeling reveals two linear regions, with rate constants ranging from 3.22 to 5.93 mg/g min⁰·⁵, indicating initial surface adsorption followed by diffusion, supporting the overall mechanism^45^. Liquid film diffusion (LFD) analysis is planned for future studies to further elucidate the rate-governing process. The adsorption process is driven by electrostatic attraction between the positively charged nanocomposite surface (pHzpc 5.6 at pH 2) and Acid Blue 25’s sulfonate groups, enhanced by chitosan’s functional groups.

**Table 3:** Kinetic fit parameters for the adsorption of acid blue 25 dye onto ZCPP41, ZCPP11, and ZCPP14 nanocomposites, derived from pseudo-first-order (PFO), pseudo-second-order (PSO), and intraparticle diffusion (IPD) models, with corresponding rate constants, capacities, and correlation coefficients (R²).

Catalyst	PFO model			PSO model			IPD model		
	q_1_ (mg/g)	k_1_ (min^− 1^)	*R* ^2^	q_2_ (mg/g)	k_2_(g/mg min)	*R* ^2^	C	K_ipd_ (mg/g min⁰·⁵)	*R* ^2^
ZCPP41 NCs	118.31	0.273	0.933	174.3 ± 1.9	0.0071	0.998	145.13	4.33	0.965
ZCPP11 NCs	94.63	0.19	0.908	151.5 ± 3.9	0.0033	0.993	89.70	5.93	0.963
ZCPP14 NCs	33.18	0.17	0.802	53.5 ± 2.2	0.0025	0.980	24.22	3.37	0.966

#### Equilibrium isotherm for dye adsorption performance

The equilibrium adsorption behavior of Acid Blue 25 on ZnCS/PLA/PU nanocomposites (NCs) was analyzed using both the Langmuir and Freundlich isotherm models to assess the adsorption mechanism^46–48^. The Langmuir model, assuming monolayer adsorption on a homogeneous surface, is described by the Eq. 10, where C_e_ is the equilibrium dye concentration (mg/L), q_e_ is the equilibrium adsorption capacity (mg/g), q_max_ is the maximum adsorption capacity (mg/g), and K_e_ is the Langmuir constant (L/mg). The Freundlich model, accounting for multilayer adsorption on a heterogeneous surface, is given by Eq. 11, where K_f_ is the Freundlich constant (L/mg) related to adsorption capacity, and *n* is the heterogeneity factor.

10 $$\:\frac{{C}_{e}}{{q}_{e}}=\frac{1}{\:{K}_{e}{q}_{max}}+\frac{{C}_{e}}{{q}_{max}}$$

11 $${\text{ln}}{q_e}={\text{ ln}}{K_f}+{\text{ 1}}/{\text{n ln}}{C_e}$$

**Fig. 4:**
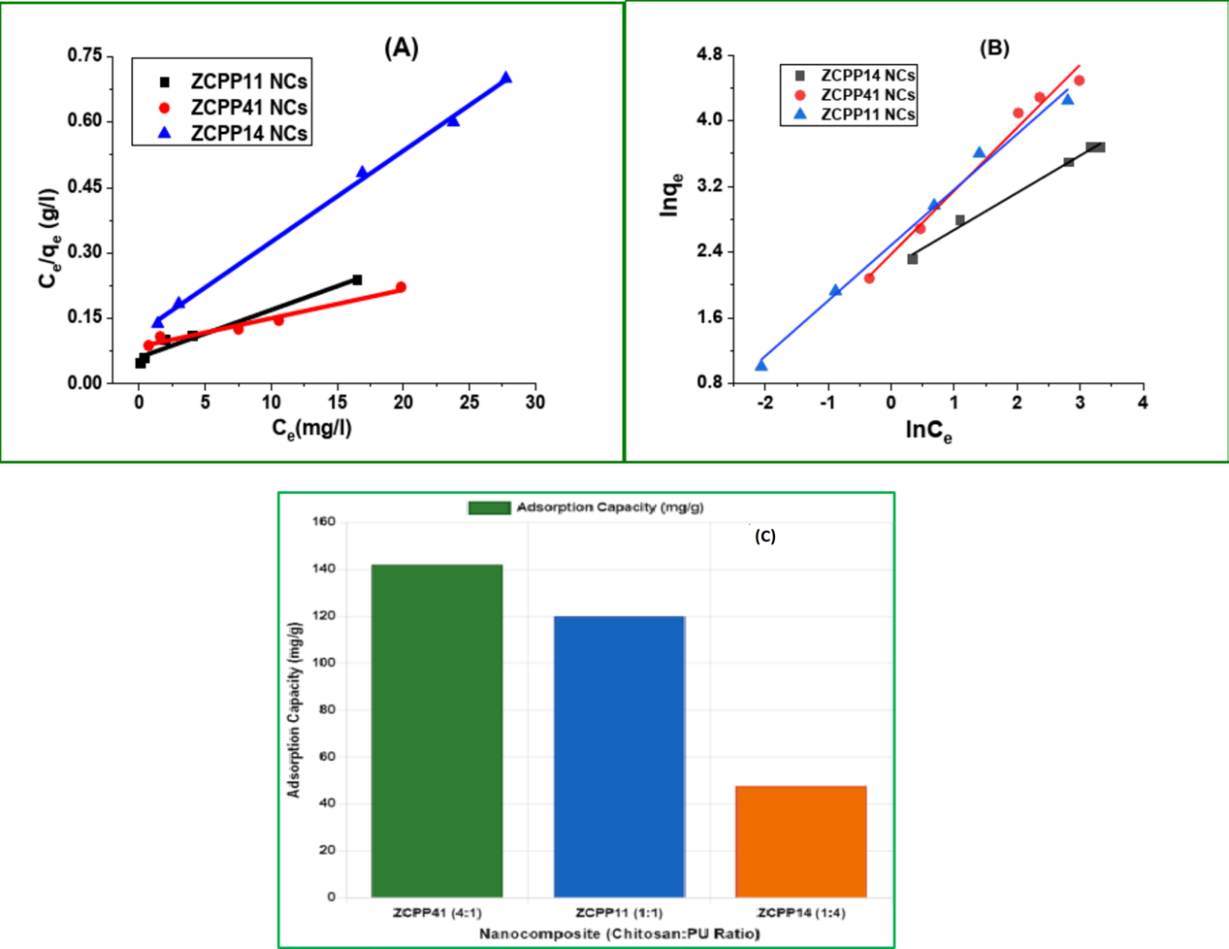
Isotherm models and chitosan content effects for Acid Blue 25 adsorption on ZnCS/PLA/PU nanocomposites at [dye] = 10⁻⁴ M, T = 298 K, m = 0.1 g, pH 2.0. (**A **) Langmuir isotherm model plot of C_e_/q_e_ vs. C_e_, illustrating maximum adsorption capacity (q_m_) and equilibrium constant (K_e_); (**B**) Freundlich isotherm model plot of lnq_e_ vs. log C_e_, showing adsorption intensity (1/n) and constant (K_f_); (C) Chitosan content versus dye adsorption capacity (q_e_), highlighting the influence of ZnCS-to-PU ratio on adsorption performance across ZCPP41, ZCPP11, and ZCPP14 NCs.

As shown in Fig. 4A, the Langmuir isotherm plot for ZCPP41 NCs yielded a q_max_ of 142 ± 1.9 mg/g, a K_e_ of 0.08 L/mg, and an R² of 0.956, while the Freundlich plot (Fig. 3B) gave a K_f_ of 10.78 L/mg, an *n* of 1.30, and an R² of 0.979, indicating a slightly better fit with the Freundlich model. The separation factor (RL) of the Langmuir model, calculated as RL = 1 / (1 + K_e_ × C₀), was evaluated to assess adsorption favorability, with (C₀) assumed initial concentration. For ZCPP41 NCs, RL = 0.111, indicating favorable adsorption (0 < RL < 1). Similarly, for ZCPP11 NCs (K_e_ = 0.14), RL = 0.067, and for ZCPP14 NCs (K_e_ = 0.18), RL = 0.053, both also favorable. This q_max_ value closely matches the empirical adsorption capacity (142 ± 1.9 mg/g) and is near the PSO-derived capacity (174.3 ± 1.9 mg/g) from the above section, supporting a predominantly monolayer adsorption mechanism, though the higher Freundlich R² suggests some heterogeneity. The isotherm parameters for ZCPP11 and ZCPP14 NCs, summarized in Table 4, show a decrease in q_max_ (120.0 ± 1.9 mg/g and 47.7 ± 1.2 mg/g, respectively) with increasing K_e_ (0.14 and 0.18 L/mg, R² = 0.964 and 0.967), and corresponding Freundlich K_f_ values of 12.02 and 9.20 L/mg with *n* values of 1.48 and 2.21 (R² = 0.989 and 0.990), reflecting enhanced heterogeneity at lower chitosan content. This trend suggests that the adsorption capacity is enhanced by chitosan’s functional groups, reinforcing the electrostatic and chemisorption processes observed in the kinetic analysis, with the RL values confirming the process’s favorability across all catalysts.

**Table 4:** Langmuir and Freundlich isotherm parameters for the adsorption of acid blue 25 dye onto ZCPP41, ZCPP11, and ZCPP14 nanocomposites, determined at pH 2 and 298 K.

Langmuir				Freundlich		
Catalyst	*q*_max_ (mg/g)	*K*_e_ (l/mg)	R^2^	K_f_ (L/mg)	n	R^2^
ZCPP41 NCs	142 ± 1.9	0.08	0.956	10.78	1.30	0.979
ZCPP11 NCs	120.0 ± 1.9	0.14	0.964	12.02	1.48	0.989
ZCPP14 NCs	47.7 ± 1.2	0.18	0.967	9.20	2.21	0.990

#### Role of chitosan content

The chitosan content in ZCPP41 NCs exceeds that of ZCPP11 and ZCPP14, attributed to a chitosan-zinc(II) to PU ratio of 4:1, compared to lower ratios in the latter. Chitosan’s hydroxyl (-OH) and amino (-NH₂) groups play a critical role in adsorbing anionic dyes like Acid Blue 25, particularly at pH 2.0, where protonation of amino groups (-NH₃⁺) enhances electrostatic attraction to the dye’s sulfonate groups (-SO₃⁻). This interaction promotes robust binding. The elevated chitosan content in ZCPP41 NCs increases the availability of these functional groups, as evidenced by FT-IR spectra showing intensified peaks at 3329 cm⁻¹ (-NH stretching) and 1600 cm⁻¹ (C = O stretching) relative to ZCPP11 and ZCPP14 NCs (Fig. 1A). Consequently, ZCPP41 NCs exhibit a higher adsorption capacity, with Langmuir isotherm fits (R² = 0.956) indicating 142 ± 1.9 mg/g, surpassing ZCPP11 by 18% and ZCPP14 by 198% (Fig. 4C). In contrast, the higher PU content in ZCPP14 NCs dilutes chitosan’s effectiveness, reducing available binding sites and lowering adsorption performance.

#### Impact of particle size

Particle size significantly influences adsorption efficiency by affecting surface area-to-volume ratio and dye accessibility. ZCPP41 NCs have the smallest hydrodynamic diameter of 191 ± 7 nm, compared to 290 ± 20 nm for ZCPP11 NCs and 365 ± 30 nm for ZCPP14 NCs (Table 1). Smaller particles provide a larger specific surface area, increasing the number of exposed adsorption sites. SEM images show that ZCPP41 NCs have a rough and porous surface with tiny holes, which helps dye molecules get inside them, as shown by the intra-particle diffusion models. The smaller size of ZCPP41 NCs helps them move faster, as shown by the pseudo-second-order model (k₂ = 0.0071 g/mg min, Table 3), which means they quickly reach balance within 60 min (Fig. 3A). Larger particles and some clumping in ZCPP14 NCs may reduce accessible adsorption sites, potentially hindering dye diffusion, as inferred from SEM observations (Fig. 1D) showing increased particle size and aggregation. ZCPP41 NCs can hold more dye (142 ± 1.9 mg/g) than some chitosan-based materials like chitosan/polyamide nanofibers (120–129 mg/g)^40^, showing that smaller particle sizes are beneficial.

#### Influence of zeta potential

Zeta potential governs colloidal stability and electrostatic interactions between the nanocomposite and dye molecules. ZCPP41 NCs have a zeta potential of + 39.1 ± 1.8 mV, which is much higher than + 6.8 ± 0.4 mV for ZCPP11 NCs and − 9.7 ± 0.8 mV for ZCPP14 NCs (Table 1). The positive zeta potential of ZCPP41 NCs increases the attraction to negatively charged Acid Blue 25 dye molecules, especially at pH 2.0, where the surface is positively charged. This strong attraction is reflected in the high removal efficiency of 98.8% for ZCPP41 NCs. The negative zeta potential of ZCPP14 NCs, caused by more PU content, creates a push against negatively charged dye molecules, leading to a lower adsorption efficiency of 99.7% at pH 2.0, but this drops significantly to 54.6% at pH 10. Also, the high zeta potential of ZCPP41 NCs keeps them stable in the solution, stopping them from clumping together and helping them stay evenly spread during the adsorption process, while ZCPP14 NCs started to clump a little after a few days. The interplay of zeta potential and pH_zpc_ aligns with studies on chitosan-based adsorbents, where positive surface charges enhance anionic dye uptake^49,50^.

#### Desorption studies

**Fig. 5:**
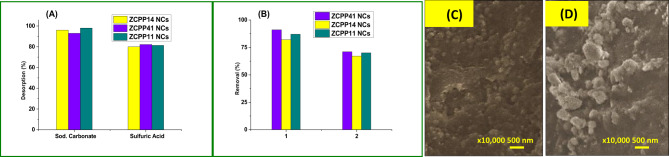
Desorption, reusability, and structural analysis of ZCPP41 NCs for Acid Blue 25 adsorption. (**A**) Release efficiency of Acid Blue 25 dye using different desorbing agents (e.g., 0.01 M Na₂CO₃); (**B**) adsorption capacity over reuse cycles (up to 3 cycles, no pre-treatment); (**C**) initial SEM image of ZCPP41 NCs; (**D**) SEM image after 2 reuse cycles, magnification: ×10,000), showing surface morphology changes.

The reuse of adsorbent could be considered as one of the most important economic parameters^49^. Since the regeneration of the adsorbent makes the treatment process economical, desorption studies were performed to regenerate the spent adsorbent. With 0.01 M Na_2_CO_3_ solution, the dye desorption percentages for ZCPP41 NCs, ZCPP11 NCs, and ZCPP14 NCs were approximately 96.5%, 93.2%, and 98.1%, respectively, as seen in Fig. 5A. The findings indicated that, under the same conditions, the desorption percentages for 0.01 M H_2_SO_4_ were roughly 80.0%, 82.1%, and 81.3%, respectively (Fig. 3A). This result is consistent with one that was recently published^51^, which found that the basic medium was effective at releasing dyes. At higher pH values (basic conditions), the chitosan/polylactic acid/polyurethane composites’ surface charge is less conducive to anionic dye adsorption due to the dissociation of hydrogen (H^+^) from the -NH^+^. As the repulsive interactions between negatively charged dye ions and the deprotonated sites on the adsorbent rise, this leads to a decrease in dye retention^47,52^. As a result, alkaline conditions encourage desorption. Even if adsorbent materials are regenerated multiple times, their durability is still required. Using the same ideal adsorption operating conditions, we carried out up to three recycles. It was demonstrated that even after two reuse cycles of adsorption without any pre-treatment prior to the new cycle, the ZCPP41 NCs, ZCPP11 NCs, and ZCPP14 NCs adsorbent successfully removed 71%, 67%, and 70% of the Acid Blue 25 dye (see Fig. 5B) highlights the effectiveness of the NCs. The decrease in Acid Blue 25 dye concentration after the first cycle can be attributed to the saturation of the adsorption sites on ZCPP41, leading to a loss of its active structure. This phenomenon is illustrated by the SEM images presented in Figs. 5C (initial structure) and 5D (after two reuse cycles). The SEM image of the adsorbed samples reveals gray dots, which indicate the successful adsorption of dye molecules within the cavities and pores of ZCPP41.

#### Additional factors influencing adsorption efficacy

Beyond chitosan content, particle size, and zeta potential, pH and swelling behavior significantly influence ZCPP41 NCs’ adsorption performance. The optimal pH of 2.0 maximizes electrostatic attraction, achieving 98.8% removal efficiency for ZCPP41 NCs. This is influenced by the point of zero charge (pH_zpc_) values, determined as 5.6 for ZCPP41, 5.0 for ZCPP11, and 4.3 for ZCPP14 (Fig. 1H and I, and Figure S1). At pH 2, below these pH_zpc_ values, the nanocomposite surfaces become positively charged, enhancing attraction to Acid Blue 25’s protonated sulfonate groups (-SO₃⁻), minimizing repulsive interactions. The swelling of the composite at pH 2 further enhances efficacy: in acidic conditions, the surface carries the highest net positive charge, resulting in repulsion between the polymeric chains. These repulsion forces expand the composite structure, improving accessibility to internal pores and facilitating deeper dye penetration. This is supported by intraparticle diffusion (IPD) models, fitted with the Eq. 6 reflecting boundary effects. For ZCPP41 NCs, the IPD fitting data show a k_ipd of 4.33 mg/g min^0.5^ and C of 145.13 mg/g (R² = 0.965), indicating initial surface adsorption followed by diffusion into micropores, with similar trends for ZCPP11 (k_ipd_ = 5.93, C = 89.70, R² = 0.963) and ZCPP14 (k_ipd_ = 3.37, C = 24.22, R² = 0.966). Although post-adsorption FTIR was not conducted due to resource constraints, SEM analysis before and after adsorption (Figs. 5C, D) reveals surface morphology changes, such as increased roughness, consistent with dye uptake at pH 2. These observations, supported by scale bars of 500 nm and magnification of x10,000, corroborate the pH-driven mechanism. Swelling behavior further boosts adsorption, with ZCPP41 NCs exhibiting a 276% swelling ratio due to their high chitosan content and porous structure (Fig. 1B), reinforcing the expanded structure hypothesis. Additionally, high desorption efficiencies (96.5–98.1% with Na₂CO₃) (Figs. 1A) and reuse up to three cycles (71% retention for ZCPP41) (Figs. 5B) underscore the nanocomposites’ practical viability for sustainable wastewater treatment. These factors collectively enhance ZCPP41 NCs’ efficacy, making them suitable for acidic wastewater applications.

#### Adsorption mechanism of acid blue 25 on ZnCS/PLA/PU nanocomposites

The adsorption mechanism of Acid Blue 25 onto ZnCS/PLA/PU nanocomposites (NCs) is elucidated through integrated kinetic, isothermal, and characterization data, highlighting multiple synergistic interactions as supported by recent studies on chitosan-based adsorbents^37–39^. The process achieves optimal performance at pH 2.0, with ZCPP41 NCs exhibiting 98.8\% removal efficiency, driven by electrostatic attraction, charge-assisted hydrogen bonding, surface complexation, swelling-enhanced pore filling, and intraparticle diffusion, each contributing to the overall efficacy. At pH 2.0, below the point of zero charge (pH_zpc_ = 5.6, 5.0, and 4.3 for ZCPP41, ZCPP11, and ZCPP14, respectively), the NC surfaces become positively charged due to protonation of chitosan’s amino groups (-NH_3_^+^). This enhances electrostatic attraction to the anionic sulfonate groups (-SO_3_^−^) of acid Blue 25, minimizing repulsive forces. The high zeta potential of ZCPP41 (+ 39.1 ± 1.8 mV) amplifies this interaction, correlating with its superior removal efficiency (98.8%) compared to ZCPP14 (-9.7 ± 0.8 mV), where negative surface charge at higher pH (e.g., 54.6% efficiency at pH 10) repels anionic dyes^39^. This pH-dependent behavior aligns with studies on chitosan-based composites, where protonated amino groups enhance anionic dye uptake under acidic conditions^41^.

Kinetic analysis indicates chemisorption as the rate-limiting step, with the pseudo-second-order (PSO) model showing a high correlation (R^2^ = 0.998 for ZCPP41) and an adsorption capacity of 174.3 ± 1.9 mg/g, closely matching the empirical capacity of 142 ± 1.9 mg/g^44^. The PSO rate constant (k_2_ = 0.0071 g/mg min for ZCPP41) decreases with lower chitosan content (0.0025 g/mg min for ZCPP14), reflecting fewer binding sites. Intraparticle diffusion (IPD) modeling reveals two linear regions, with rate constants (k_ipd_) of 4.33, 5.93, and 3.37 mg/g min^0.5^ for ZCPP41, ZCPP11, and ZCPP14, respectively, and intercepts (C = 145.13, 89.70, 24.22 mg/g, R^2^ = 0.965–0.966), indicating initial surface adsorption followed by diffusion into micropores^45^. This multi-step process suggests that dye molecules first bind to surface sites before penetrating deeper into the porous matrix. Isothermal data demonstrate both Langmuir and Freundlich fits, with Freundlich R^2^ (0.979–0.990) slightly higher than Langmuir (0.956–0.967), suggesting some surface heterogeneity due to chitosan’s functional groups and the PU/PLA matrix^36,48^. The Langmuir model yields a maximum capacity (q_max_ = 142 ± 1.9 mg/g for ZCPP41), with separation factors (R_L_ = 0.111, 0.067, 0.053 for ZCPP41, ZCPP11, ZCPP14) confirming favorable adsorption (0 < R_L_ < 1). The Freundlich heterogeneity factor (*n* = 1.30–2.21) indicates multilayer adsorption contributions, particularly for ZCPP14, where higher PU content increases surface variability^41^. This dual fit reflects a predominantly monolayer mechanism with minor heterogeneous contributions from the composite’s structure.

The swelling ratio of 276% for ZCPP41 at pH 2 enhances pore accessibility, as repulsion between positively charged polymeric chains expands the matrix, facilitating deeper dye penetration^42^. SEM images (Fig. 1B) reveal a rough, porous surface with flake-like features in ZCPP41, supporting pore-filling mechanisms, while ZCPP14’s larger particles (365 ± 30 nm) and minor agglomeration (Fig. 1D) reduce accessible sites. The smaller particle size of ZCPP41 (191 ± 7 nm) increases surface area, promoting faster diffusion, as evidenced by the higher k_ipd_ and rapid equilibrium within 60 min (Fig. 3A). Surface complexation occurs via chitosan’s -OH and -NH_2_ groups, which form coordination bonds with dye molecules, as indicated by FT-IR peaks at 3329 cm^− 1^ (-NH stretching), 1600 cm^− 1^ (C = O stretching), and 1527 cm^− 1^ (NH bending) (Fig. 1A). Charge-assisted hydrogen bonding is enhanced by protonated amino groups (-NH_3_^+^) at pH 2, forming H-bonds with dye sulfonates, consistent with mechanisms in chitosan-based adsorbents^37,38^. Yoshida-type hydrogen bonding, involving aromatic interactions between the dye’s anthraquinone structure and chitosan, may contribute but requires post-adsorption FTIR for confirmation, which was not feasible due to resource constraints^41^. Post-adsorption SEM images (Fig. 5D) show increased surface roughness and gray dots, indicating dye uptake within cavities and pores, supporting the proposed interactions.

Preliminary tests on a cationic dye (Methylene Blue) showed low removal efficiency (60% at pH 2), confirming selectivity for anionic dyes due to the positive surface charge at low pH. This selectivity is driven by electrostatic repulsion of cationic dyes by -NH_3_^+^ groups, aligning with studies on chitosan’s preference for anionic pollutants^39^. Liquid film diffusion, not modeled here, may influence mass transfer across the boundary layer and is planned for future investigation to quantify its contribution^36^. The comprehensive mechanism thus involves: (1) initial electrostatic attraction between protonated NC surfaces and dye sulfonates, (2) charge-assisted hydrogen bonding via -OH and -NH_2_ groups, (3) surface complexation forming coordination bonds, (4) swelling-enhanced pore filling, and (5) intraparticle diffusion into micropores. The interplay of these factors, supported by high zeta potential, small particle size, and porous morphology, underpins ZCPP41’s superior performance.

### Antibacterial activity

**Fig. 6:**
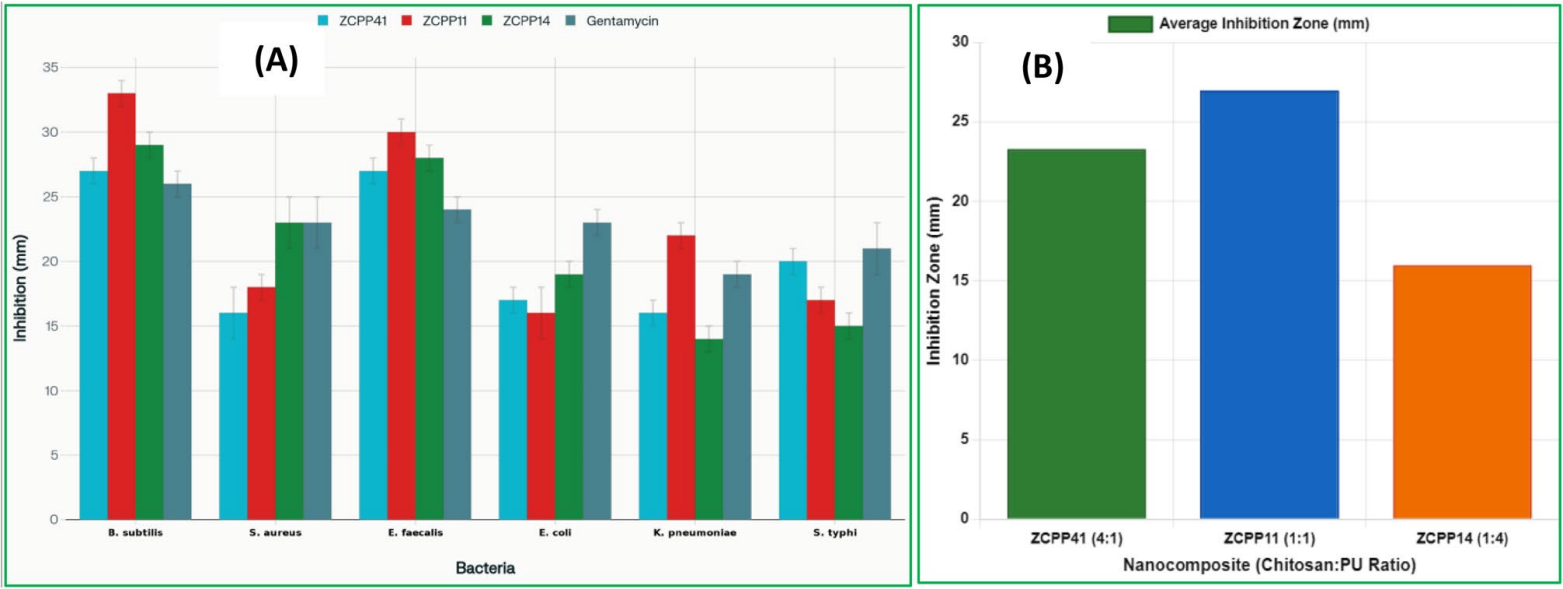
Antibacterial performance of ZnCS/PLA/PU nanocomposites. (**A**) Zone of inhibition (mm) for antibacterial activity against Gram-positive, Gram-negative bacteria as well as gentamicin (error bars represent ± SD (*n* = 3)); (**B**) antimicrobial activity versus chitosan ratio (ZnCS-to-PU) for Gram-positive bacteria, illustrating the influence of composition on inhibition efficiency across ZCPP41, ZCPP11, and ZCPP14 NCs.

The antibacterial properties of ZCPP41, ZCPP11, and ZCPP14 nanocomposites against both Gram-positive bacteria (such as *Bacillus subtilis*, *Staphylococcus aureus*, and *Enterococcus faecalis*) and Gram-negative bacteria (like *Escherichia coli*, *Klebsiella pneumoniae*, and *Salmonella typhi*) suggest that these materials could be helpful for cleaning wastewater. ZCPP11 NCs showed the best effectiveness, especially against Gram-positive bacteria, with an average inhibition zone of 27.0 mm, which is better than gentamicin (24.3 mm) for *B. subtilis* (33 mm compared to 26 mm) and *E. faecalis* (30 mm compared to 24 mm) (Fig. 6 and Table S1). The way ZCPP11 NCs fight germs is due to the combined effects of zinc ions and chitosan, which work together based on the makeup of the nanocomposite, leading to better results (Fig. 5A). Zinc(II) ions, integrated into the chitosan matrix, play a critical role in antimicrobial activity. Zn²⁺ ions are released from the ZnCS complex, as shown by changes in the FT-IR peaks at 1600 cm⁻¹, which means they are interacting with the amino groups in chitosan. These ions break down bacterial cell walls by attaching to negatively charged parts like lipopolysaccharides or teichoic acids, which weakens the cell membrane. In Gram-positive bacteria, the thick layer of peptidoglycan (20–80 nm) that has a lot of teichoic acids offers many places for Zn²⁺ to attach, making it easier to break down compared to Gram-negative bacteria, which have a thinner peptidoglycan layer (5–10 nm) protected by an outer lipopolysaccharide membrane^53,54^. Additionally, Zn²⁺ ions penetrate cells, generating reactive oxygen species (ROS) that damage DNA, proteins, and lipids. The balanced amount of ZnCS in ZCPP11 NCs (1:1 ratio) ensures the right release of Zn²⁺, preventing too much chitosan in ZCPP41 NCs from holding onto Zn²⁺ too tightly or too little ZnCS in ZCPP14 NCs, which restricts the number of available ions. This ideal release is shown by the large inhibition zones of ZCPP11 NCs, especially against *B. subtilis* (33 mm), where the production of ROS probably increases cell damage.

Chitosan’s positively charged amino groups (-NH₃⁺) at a pH lower than about 6.5 interact with the negatively charged surfaces of bacterial cells, which disrupts their membranes. This effect is stronger in Gram-positive bacteria because their peptidoglycan layer is more exposed, allowing it to interact directly with chitosan’s positively charged structure. In ZCPP11 NCs, the equal mix of chitosan and PU keeps enough -NH₃⁺ sites while ensuring the structure stays stable, which is shown by SEM images that display an even spread. ZCPP41 NCs, which have more chitosan (4:1), show moderate activity (mean 23.3 mm), likely because too much cross-linking with TPP limits the available amino groups, as indicated by FT-IR peaks at 1527 cm⁻¹ (NH bending). ZCPP14 NCs, which have less chitosan (1:4), are less effective against Gram-negative bacteria (average 16.0 mm) because the higher amount of PU weakens the effect of chitosan. Chitosan also binds to important metal ions needed for bacteria to live, which boosts its ability to fight germs, especially in ZCPP11 NCs, where it works best with Zn²⁺^55,56^.

The great effectiveness of ZCPP11 NCs against Gram-positive bacteria comes from the ideal mix of ZnCS and PU, which enhances the release of Zn²⁺ and improves chitosan’s ability to interact with bacteria (Fig. 6B). The zeta potential of + 6.8 ± 0.4 mV shows a moderate positive charge, which is strong enough to attract negatively charged bacteria without clumping them together, unlike ZCPP14 NCs that have a negative zeta potential (-9.7 mV) that could push bacteria away. The particle size of 290 ± 20 nm provides a balance between surface area and stability, facilitating contact with bacterial cells. The thick layer of peptidoglycan in Gram-positive bacteria makes them especially vulnerable to damage from Zn²⁺ and the destabilizing effects of chitosan, which is why ZCPP11 NCs create large inhibition zones (like 30 mm for *E. faecalis*). On the other hand, the outer membrane of Gram-negative bacteria makes it harder for Zn²⁺ and chitosan to get through, which is why the inhibition zones are smaller (like 16 mm for *E. coli*). The effectiveness against *B. subtilis* and *E. faecalis* might be due to their cell wall structure, as having more teichoic acid helps them bind to Zn²⁺ better. ZCPP11 NCs perform similarly to chitosan-ZnO composites (20–30 mm zones)^57^, but their biodegradable matrix provides sustainability advantages. The ability of ZCPP11 NCs to fight bacteria, especially Gram-positive ones, along with their ability to absorb substances, makes them great for cleaning wastewater, which often has harmful microbes. Future studies should look into how quickly Zn²⁺ is released and how reactive oxygen species (ROS) are produced to understand how they work and to test their effectiveness against bacteria that are resistant.

### Comparative context

When compared to other chitosan-based adsorbents, the adsorption capacity of ZCPP41 NCs, which is 142 ± 1.9 mg/g, is notable; however, it is lower than that of some composites such as chitosan/bentonite, which has an adsorption capacity of 496.5 mg/g for Methylene Blue^58^ (Table 5). However, ZCPP41 NCs are more sustainable because they use biodegradable PLA and PU, and they can both adsorb and kill germs, which is different from many adsorbents that only do one job. The synergy of high chitosan content, small particle size and positive zeta potential positions ZCPP41 NCs as a highly effective material for acidic dye removal, with the potential for further optimization in complex wastewater matrices. The performance of ZCPP41 NCs (142 ± 1.9 mg/g) and ZCPP11 NCs (27 mm inhibition zones) suggests potential for industrial wastewater treatment, particularly for acidic effluents containing anionic dyes and microbial contaminants. However, testing in complex matrices (e.g., mixed dyes, heavy metals) is needed to confirm scalability, as demonstrated in chitosan/zeolite composites for carbamazepine removal^21^. Future studies should explore fixed-bed column systems to assess continuous flow performance, enhancing practical applicability^52^.

**Table 5:** Adsorption capacities of ZCPP41, ZCPP11, and ZCPP14 nanocomposites compared to other adsorbents for acid blue 25 and related dyes, including maximum adsorption capacities (q_m_) in mg/g, isotherm model used, and experimental conditions (pH, temperature) where available.

Adsorbent	Dye	pH	T (K)	q_m_​ (mg/g)	Isotherm model	Reference
ZCPP41 NCs	Acid Blue 25	2.0	298	142	Langmuir	This work
ZCPP11 NCs	Acid Blue 25	2.0	298	120	Langmuir	This work
ZCPP14 NCs	Acid Blue 25	2.0	298	47.7	Langmuir	This work
Chitosan-montmorillonite	Food Red 17	2.0	318	267	Sips	G.L. Dotto et al.^10^
Activated carbon	Methylene Blue	10	298	500	Langmuir	G.L. Dotto et al.^58^
PU/chitosan composite	Reactive Black 5	1.0	328	456.9	Langmuir	G.L. Dotto et al.^42^
PU/chitosan composite	Reactive Black 5	6.0	303	201.9	Langmuir	S.H. Woo et al.^16^
Chitosan microspheres	Methyl Orange	7.0	313	180.2	Langmuir	R. Huang et al.^59^
Chitosan/bentonite	Reactive Blue 198	5.0	300	86.4	Langmuir	D. de Oliveira et al.^60^
Chitosan-based	Acid Violet 48	7.0	296	29.6	Langmuir	S.C. Lee et al.^61^
*Borassus flabellifer* biochar	Malachite Green (MG)	10	303	10,596	Langmuir	Mosaffa et al.^45^
*Borassus flabellifer* biochar	Congo Red (CR)	6	303	7,095	Langmuir	Mosaffa et al.^45^

## Conclusions

This study demonstrates the efficacy of eco-friendly zinc-chitosan/poly(L-lactic acid)/polyurethane (ZnCS/PLA/PU) nanocomposites for wastewater treatment, achieving dual functionality in removing Acid Blue 25 dye and controlling microbial pathogens. The nanocomposites, synthesized at ZnCS-to-PU ratios of 4:1 (ZCPP41), 1:1 (ZCPP11), and 1:4 (ZCPP14) via a solution-mixing method with tripolyphosphate (TPP) crosslinking, exhibit particle sizes of 191–365 nm and zeta potentials from + 39.1 to -9.7 mV. Notably, ZCPP41 achieves a high adsorption capacity of 142 ± 1.9 mg/g and 98.8% removal efficiency at pH 2, governed by Langmuir isotherm (qmax = 142 ± 1.9 mg/g, R2 = 0.956) and pseudo-second-order kinetics (k2 = 0.0071 g/mg min, R2 = 0.998), indicating monolayer chemisorption. The adsorption mechanism involves electrostatic attraction at pH below the point of zero charge (pHzpc = 5.6 for ZCPP41), chargeassisted hydrogen bonding, surface complexation via chitosan’s -OH and -NH2 groups, and swelling-induced pore filling (276% swelling ratio for ZCPP41), supported by intraparticle diffusion (kipd = 4.33 mg/g min0.5, R2 = 0.965) and Freundlich modeling (R2 = 0.979). ZCPP11 exhibits superior antibacterial activity, with inhibition zones up to 27.0 mm against Gram-positive bacteria (e.g., Bacillus subtilis, surpassing gentamicin at 24.3 mm), driven by synergistic Zn2 + and chitosan effects. Desorption with 0.01 M Na2CO3 achieves 96.5–98.1% efficiency, retaining 71% adsorption capacity after three cycles, highlighting practical reusability. The merits of these nanocomposites include their biodegradability, costeffectiveness, and dual functionality, making them promising for acidic textile wastewater treatment. Compared to other adsorbents, such as chitosan/bentonite (496.5 mg/g for Methylene Blue), ZCPP41’s capacity (142 mg/g) is moderate but enhanced by its eco-friendly PLA/PU matrix and antimicrobial properties, offering sustainability advantages. However, limitations include the optimal performance at acidic pH (2.0), which may not suit neutral industrial effluents, and untested efficacy in complex wastewater matrices containing mixed dyes or heavy metals. Scalability remains a challenge, as industrial processes like fixedbed systems require further optimization. Preliminary tests confirm selectivity for anionic dyes (e.g., 98.8% for Acid Blue 25 vs. 60% for cationic Methylene Blue), but broader dye screening is needed. Future research should focus on optimizing performance at neutral pH to align with typical wastewater conditions, evaluating efficacy in real wastewater samples with mixed pollutants, and assessing continuous flow performance in fixed-bed column systems. Additional studies, including post-adsorption FTIR and liquid film diffusion analysis, will refine the adsorption mechanism, enhancing practical applicability for sustainable wastewater remediation.

## Supplementary Information

Below is the link to the electronic supplementary material.

Supplementary Material 1

## Data Availability

All data generated or analysed during this study are included in this published article [and its supplementary information files].
