# Supplementary material for: Eco-friendly zinc-chitosan/poly(l-lactic acid)/polyurethane nanocomposites for dye removal and antimicrobial wastewater treatment
Source: Sci Rep. 2025 Sep 15;15:32534. doi: 10.1038/s41598-025-18431-x (PMC12436595; doi:10.1038/s41598-025-18431-x)
Supplement: Supplementary file 1 — Supplementary Material 1 [file 41598_2025_18431_MOESM1_ESM.docx]

**Eco-Friendly Chitosan-Based ZnCS/PLA/PU Nanocomposites for Dye Removal and Antimicrobial Wastewater Treatment**

| \|  \| \| --- \|   **Figure S1.** Point of zero charge (pHzpc) determination for ZCPP11 nanocomposite sorbent, illustrating surface charge properties over a pH range of 2–12, with pHzpc ≈ 5.6 indicating the pH at which the net surface charge is zero Point of zero charge (pHzpc) values for ZCPP11 sorbent, indicating surface charge properties.   \|  \|  \| \| --- \| --- \| \| (A) \| (B) \|   **Figure S2.** Influence of operational parameters on Acid Blue 25 adsorption by ZCPP41, ZCPP11, and ZCPP14 nanocomposites. (A) Effect of catalyst dose on adsorption efficiency, evaluated over a range of 0.05–0.25 g; (B) Effect of temperature on adsorption capacity, assessed from 304 to 319 K.   |
| --- | --- | --- | --- | --- | --- |

**Figure S3.** Pseudo-first-order (PFO) kinetic fits for the adsorption of Acid Blue 25 dye onto ZCPP41, ZCPP11, and ZCPP14 nanocomposites, conducted at a dye concentration of 10⁻⁴ M, 304 K, and pH 2.0, with plots of ln(q_e_-q_t_) vs time.

**Table S1**. Antibacterial activity of polyurethane (PU)/polylactic acid (PLA)/chitosan-zinc (II) (**ZCPP**) nanocomposites in a three ratios of chitosan-zinc(II) to PU, 4:1 (**ZCPP41**), 1:1 (**ZCPP11)** and 1:4 (**ZCPP14**). Zone of inhibition (mm) values represents the mean (±) standard error of three independent antibacterial assays and zone of inhibition was measured as mm in diameter).

| Test samples | Gram-positive bacteria | | |  | Gram-negative bacteria | | |
| --- | --- | --- | --- | --- | --- | --- | --- |
|  | *B. subtilis* | *S. aureus* | *E. faecalis* |  | *E. coli* | *K. pneumoniae* | *S. typhi* |
| ZCPP41 NCs | 27±1 | 16±2 | 27±1 |  | 17±1 | 16±1 | 20±1 |
| ZCPP11 NCs | 33±1 | 18±1 | 30±1 |  | 16±2 | 22±1 | 17±1 |
| ZCPP14 NCs | 29±1 | 23±2 | 28±1 |  | 19±1 | 14±1 | 15±1 |
| Gentamycin | 26±1 | 23±2 | 24±1 |  | 23±1 | 19±1 | 21±2 |
